# Self-Guided Psychological Treatment for Depressive Symptoms: A Meta-Analysis

**DOI:** 10.1371/journal.pone.0021274

**Published:** 2011-06-21

**Authors:** Pim Cuijpers, Tara Donker, Robert Johansson, David C. Mohr, Annemieke van Straten, Gerhard Andersson

**Affiliations:** 1 Department of Clinical Psychology, VU University Amsterdam, Amsterdam, The Netherlands; 2 Department of Behavioural Sciences and Learning, Swedish Institute for Disability Research, Linköping University, Linköping, Sweden; 3 Department of Preventive Medicine, Northwestern University, Chicago, Illinois, United States of America; 4 Psychiatry Section, Department of Clinical Neuroscience, Karolinska Institutet, Stockholm, Sweden; RAND Corporation, United States of America

## Abstract

**Background:**

A number of trials have examined the effects of self-guided psychological intervention, without any contact between the participants and a therapist or coach. The results and sizes of these trials have been mixed. This is the first quantitative meta-analysis, aimed at organizing and evaluating the literature, and estimating effect size.

**Method:**

We conducted systematic literature searches in PubMed, PsycINFO and Embase up to January 2010, and identified additional studies through earlier meta-analyses, and the references of included studies. We identified seven randomized controlled trials that met our inclusion criteria, with a total of 1,362 respondents. The overall quality of the studies was high. A post-hoc power calculation showed that the studies had sufficient statistical power to detect an effect size of *d* = 0.19.

**Results:**

The overall mean effect size indicating the difference between self-guided psychological treatment and control groups at post-test was *d* = 0.28 (p<0.001), which corresponds to a NNT of 6.41. At 4 to 12 months follow-up the effect size was *d* = 0.23. There was no indication for significant publication bias.

**Conclusions:**

We found evidence that self-guided psychological treatment has a small but significant effect on participants with increased levels of depressive symptomatology.

## Introduction

It is well-established that self-help interventions can have positive effects on symptoms of depression [1]–[4], anxiety disorders [5], [6], sleep problems [7], headache [8], and many other health-related problems [9]. Most research on self-help interventions has focused on guided self-help, in which a professional therapist or coach supports the patient when working through the treatment. A considerable number of studies and meta-analyses have found that guided self-help for depression is effective compared to untreated control conditions [1]–[3], [5] and that it may be as effective as face-to-face treatments [10].

Whether self-guided psychological treatment without therapist support is also effective has been examined in a considerable number of studies, but the results are mixed. Some studies do find small, but statistically significant effects [11], [12], whereas others do not find any effects [13], [14] If the effects of self-guided psychological treatments are small, however, it is very well possible that individual studies do not have sufficient statistical power to detect such small effects. With the help of meta-analyses with sufficient statistical power it may be possible to detect reliable small effects.

A few earlier meta-analyses have examined the effects of self-guided psychological treatment. One meta-analysis was focused on Internet-based treatments of depression and anxiety, and found a small but significant effect [6]. However, this study was aimed at both depression and anxiety, and included only two studies on self-guided psychological treatment for depression. Another meta-analysis was aimed at therapist guided self-help as well as self-guided psychological treatment [1], and included a considerable number of studies. However, that study was aimed at identifying predictors of outcome of self-help treatments and did not examine nor report outcomes for self-guided therapy. Moreover, the study did not examine heterogeneity in studies on self-guided psychological treatment, or possible moderators and publication bias. It also included studies in which participants had high levels of stress or anxiety. Finally, a recent meta-analysis on computerized treatments for depression included studies on guided and unguided computerized treatments [15], but did not include other self-help studies and at least three additional studies have been published following the search period for that meta-analysis.

In conclusion, self-guided psychological treatment has been stimulated recently by the growth of the Internet, and many new studies on Internet-based self-guided psychological treatment have been conducted in the last few years. Moreover, while there have been studies and meta-analyses on guided self-help treatments there has been no systematic review or meta-analysis on self-guided unsupported psychological treatment for patients with depressive symptoms. The aim of this study was to conduct such a meta-analysis.

## Methods

### Identification and selection of studies

We used an existing database of randomized controlled trials on the psychological treatments of depression. This database has been described in detail elsewhere [16], and has been used in a series of 25 earlier published meta-analyses (www.evidencebasedpsychotherapies.org). The database is continuously updated through comprehensive literature searches (from 1966 to January 2010). In these searches we examined 10,346 abstracts in PubMed (1,831 abstracts), PsycINFO (2,943), Embase (3,087) and the Cochrane Central Register of Controlled Trials (2,485). These abstracts were identified by combining terms indicative of psychological treatment and depression (both MeSH-terms and text words). Details of the search strings are presented in a previous study [16]. The full search string for PubMed is presented in Appendix S1. We also checked the primary studies from 42 previous meta-analyses of psychological treatment for depression to secure that no published studies had been missed (www.evidencebasedpsychotherapies.org).

We included studies examining the effects of a self-guided psychological treatment on adults with a depressive disorder according to a diagnostic interview or an elevated level of depressive symptomatology (as indicated by a score above a cut-off score on a validated self-report depression scale like the Beck Depression Inventory). We included only randomized trials in which a self-guided psychological treatment was compared with a control condition (waiting-list, care-as-usual, or placebo). Therapies had to be fully self-guided, so without any contact with a therapist, coach or research assistant during the treatment. This meant that studies in which telephone support was given were excluded, even if the support was not of a therapeutic nature [17]. The selection of the study was conducted by the first author.

We excluded studies in which participants did not have to have some level of depressive symptoms at baseline [18]–[20], studies aimed at stress reduction [21]–[23], and studies that also included patients with anxiety disorders but did not report separate outcomes for the depressed subsample [24], [25]. We also excluded studies on children and adolescents below 18 years of age and studies on inpatients. Comorbid general medical or psychiatric disorders were not used as an exclusion criterion. No language restrictions were applied.

Data abstraction from the studies was conducted by the first author (PC), and checked by the fifth author (AvS).

### Quality assessment

We assessed the validity of included studies using four criteria of the ‘Risk of bias’ assessment tool, developed by the Cochrane Collaboration [26]. This tool assesses possible sources of bias in randomized trials: Sequence generation (the method used to generate the allocation sequence is given in sufficient detail to allow an assessment of whether it should produce comparable groups); allocation concealment (the method used to conceal the allocation sequence in sufficient detail to determine whether intervention allocations could have been foreseen in advance of, or during, enrolment); blinding of participants, personnel and outcome assessors (all measures used to blind study participants and personnel from knowledge of which intervention a participant received); Incomplete outcome data (assessment of the completeness of outcome data for each main outcome and whether all randomized patients were included in the analyses). The quality assessment was conducted by two independent reviewers (PC and AvS), and disagreements were solved by discussion.

### Meta-analyses

For each comparison between self-guided psychological treatment and a control group, we calculated the effect size indicating the difference between the two groups at post-test (Cohen's *d* or standardized mean difference), and the 95% confidence intervals of the effect sizes. Effect sizes were calculated by subtracting (at post-test) the average score of the self-guided psychological treatment group from the average score of the control group, and dividing the result by the pooled standard deviations of the two groups. Effect sizes of 0.8 can be assumed to be large, 0.5 moderate and 0.2 small [27].

In the calculations of effect sizes we only used those instruments that explicitly measured symptoms of depression. None of the studies used more than one instrument to measure depression. All studies reported means and standard deviations at post-test which allowed us to calculate effect sizes directly, and we did not have to use other statistics to calculate effect sizes (e.g., transformations of *p*-values).

To calculate pooled mean effect sizes, we used the computer program Comprehensive Meta-Analysis (version 2.2.021). As we expected considerable heterogeneity among the studies, we decided to calculate mean effect sizes using a random effects model. In the random effects model it is assumed that the included studies are drawn from ‘populations’ of studies that differ from each other systematically (heterogeneity). In this model, the effect sizes resulting from included studies not only differ because of the random error within studies (as in the fixed effects model), but also because of true variation in effect size from one study to the next.

As the standardized mean difference is not easy to interpret from a clinical point of view and so we also calculated the numbers-needed-to-be-treated (NNT), using the formulae provided by Kraemer and Kupfer [28]. The NNT indicates the number of patients that have to be treated in order to generate an additional positive outcome in one of them [29].

As a test of homogeneity of effect sizes, we calculated the *I^2^*-statistic which is an indicator of heterogeneity in percentages. A value of 0% indicates no observed heterogeneity, and larger values show increasing heterogeneity, with 25% as low, 50% as moderate, and 75% as high heterogeneity [30]. We also calculated the Q-statistic, but only report whether this was significant or not.

Subgroup analyses were conducted according to the mixed effect model. In this model, studies within subgroups are pooled with the random effects model, while tests for significant differences between subgroups are conducted with the fixed effects model. For continuous variables, we used meta-regression analyses to test whether there was a significant relationship between the continuous variable and the effect size, as indicated with a Z-value and an associated *p*-value.

Publication bias was tested by inspecting the funnel plot on primary outcome measures, and by Duval and Tweedie's trim and fill procedure [31], which yields an estimate of the effect size after the publication bias has been taken into account (as implemented in Comprehensive Meta-analysis, version 2.2.021).

We did not publish a review protocol for this meta-analysis.

### Power calculation

Based on earlier meta-analyses we assumed that the effect sizes of self-guided psychological treatment were small. Therefore we decided to conduct a power calculation that allowed us to assess whether the included studies had sufficient statistical power to detect such small effect sizes. In an earlier meta-analysis of internet-based self-help therapies [6], we found that effect size for self-guided psychological treatment was *d* = 0.26 and a strickingly similar effect size of *d* = 0.25 was found in our meta-analysis on unguided computerized treatments [15]. We wanted to have sufficient statistical power in our meta-analysis to be able to detect such a small effect size.

We conducted a power calculation according to the procedures described by Bohrenstein and colleagues [32]. The number of randomized patients is typically large in studies on self-guided psychological treatment (because no therapist is involved and some studies are even fully automated, including inclusion and randomization [33]. A power calculation indicated that we would need to include at least five studies with a mean sample size of 200 (100 participants per condition), to be able to detect an effect size of *d* = 0.26 (conservatively assuming a high level of between-study variance, τ2, a statistical power of 0.80, and a significance level, alpha, of .05). Alternatively, we would need seven studies with 150 participants each to detect an effect size of *d* = 0.26, or ten studies with 100 participants.

## Results

### Selection and inclusion of studies

**Figure 1:**
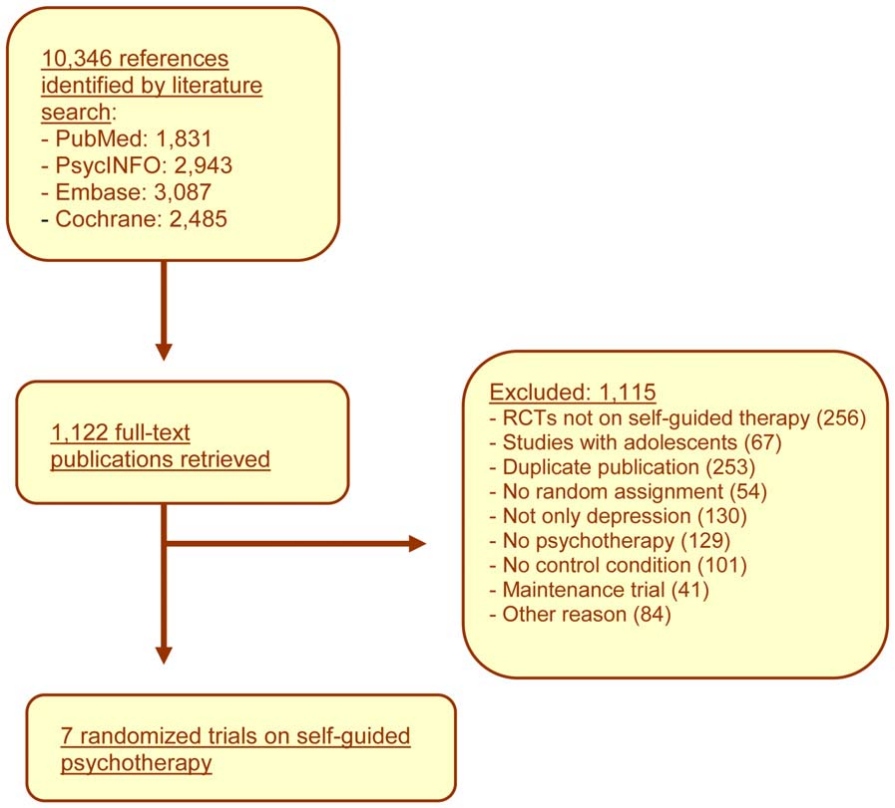
Flowchart of inclusion of studies.

In Figure 1, a flowchart describing the inclusion of studies is presented. A total of 10,346 abstracts were examined, of 1,122 the full texts were retrieved, of which 879 were excluded. A total of 263 trials were identified and included in our database (www.evidencebasedpsychotherapies.org). Seven trials examined the effects of self-guided psychological treatment, met our inclusion criteria, and were included in the current meta-analysis [11]–[14], [34]–[36].

### Characteristics of included studies

The seven studies included a total of 1,362 respondents (789 in the self-guided psychological treatment conditions and 573 in the control conditions). Selected characteristics of the studies are presented in Table 1.

**Table 1:** Selected characteristics of studies examining the effects of self-guided psychological treatment (SGP) for adult depression.

	*Recruitment*	*Diagnosis*	*% ♀*	*Mean age*	*Conditions*	*N* a)	*Self-guided therapy*	*Type* b)	*Outcome measure*	*C*	*Attrition*
Clarke, 2002	Members of an HMO	A recorded diagnosis of depression	73.6	43.3	1. SGP	107	7 chapters of CBT, based on cognitive restructuring	I	CES-D	US	34.8
					2. CAU	116					
Clarke, 2005	Members of an HMO	A recorded diagnosis of depression	72.0	50.3	1. SGP+mails	54	7 chapters of CBT, based on cognitive restructuring	I	CES-D	US	28.0
					2. SH+phonec)	67					
					3. CAU	79					
Clarke, 2009	Members of an HMO, age 18–24	A recorded diagnosis of depression	79.0	22.6	1. SGP	56	4 main sections of CBT, including psychoeducation, mood monitoring and cognitive and behavioral techniques	I	PHQ-8	US	36.9
					2. CAU	53					
De Graaf, 2009	General population	BDI-II≥16	52.0	44.3	1. SGP	100	8 CBT sessions with cognitive and behavioral techniques	I	BDI-II	NL	5.4
					2. Advise to visit GP	103					
					3. SGP+advise	100					
Meyer, 2009	General population	Self-defined depression	76.0	34.8	1. SGP	320	10 CBT modules of behavioral and cognitive techniques, mindfulness, relaxation, exercise and lifestyle, and psychoeducation.	I	BDI	GE	45.5
					2. WL	76					
Salkovskis, 2006	Depressed GP patients	MDD (DSM-IV; SCID)+BDI≥10	78.3	39.2	1. SGP	50	Personalized series of booklets about subjects such as medication, activity levels, stress, relationship problems, suicidal ideation.	B	BDI	UK	19.8
					2. CAU	46					
Spek, 2007	Older adults from general population	EDS>12, no depressive disorder (CIDI)	63.5	55.0	1. SGP	102	8 CBT sessions with cognitive and behavioral techniques	I	BDI-II	NL	38.1
					2. Group CBTd)	99					
					3. WL	100					

a) Number of randomized participants.

b) In this column an I indicates Internet-based treatment and B indicates a self-help book.

c) Because this condition included brief phone calls with respondents, it was not included in the analyses.

d) This condition was not included in the analyses.

Abbreviations:

HMO: health maintenance organization;

In only one study the presence of a depressive disorder was established with a diagnostic interview. In the remaining studies, patients had to score above a cut-off on a self-report depression scale (three studies), or patients had to have a depressive disorder according to the records of the HMO which organised the intervention (three studies).

All interventions were based on cognitive behavioural techniques. Six of the seven studies were internet-based while one study used self-help books. In four studies the intervention was compared with a care-as-usual control condition, while two studies used a waiting list control group (in the remaining study the people in the control condition were advised to contact their general practitioner). Four studies used the Beck Depression Inventory (BDI) or BDI-II as outcome measure, two used the Center for Epidemiological Stu1dies Depression scale (CES-D), and one used the Patient Health Questionnaire (PHQ-8).

In four studies there was no personal contact between the patients and the researchers, while in three studies there was personal contact at baseline (usually for the administration of questionnaires). The attrition in the studies (the percentage of participants that did not fill in the post-test questionnaires) ranged from 5.4% to 45.5%. Three studies were conducted in the United States and four in Europe (two in the Netherlands, one in Germany and one in the United Kingdom).

### Quality of included studies

The methodological quality of the studies was acceptable. In all studies the allocation sequence was generated adequately, the allocation was adequately concealed, and incomplete outcome data were adequately addressed, (in all studies intention-to-treat analyses were conducted with all randomized subjects being included in the analyses). All studies only used self-report outcome measures, and because participants were not blinded this may have introduced bias.

### Effects of self-guided psychological treatment compared to control conditions

**Figure 2:**
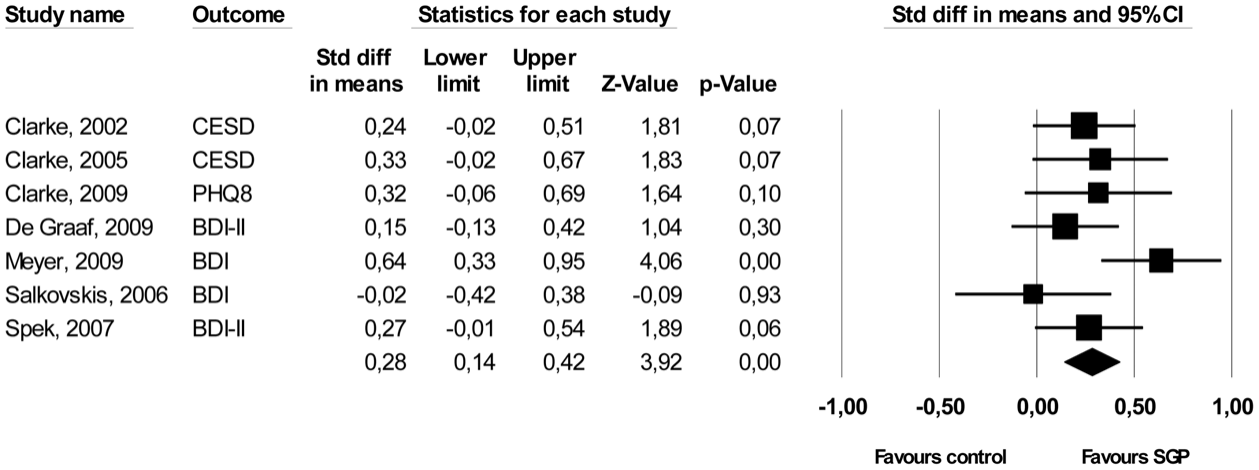
Standardized effect sizes of self-guided psychological treatment for adult depression: Cohen's d.

The overall mean effect size indicating the difference between self-guided psychological treatment and control groups at post-test was *d* = 0.28 (95% CI: 0.14,0.42; p<0.001), which corresponds to a NNT of 6.41. The effect sizes of the individual studies ranged from d = −0.02 to 0.64, with five of the six studies having a positive effect on depression. Heterogeneity was low (*I^2^* = 28.73%) and not significant. These results are summarized in Table 2, and in Figure 2. The between-study variance (τ2) was small (0.01), resulting in considerable statistical power. A post-hoc power calculation showed that our set of studies had sufficient statistical power to detect a significant effect size of *d* = 0.19.

**Table 2:** Meta-analyses of studies examining the effects of psychological treatments for depressed inpatients: Effect sizes.

*Study*		*N_comp_*	*d*	*95% CI*	*Z*	*I^2^* a)	*p* b)	*NNT*
▪ All studies		7	0.28	0.14, 0.42	3.92***	28.73		6.41
▪ Follow-up (4–6 months)		3	0.18	−0.01, 0.37	1.83	0		9.80
▪ Follow-up (8–12 months)		3	0.27	0.10, 0.44	3.07**	0		6.58
Subgroup analyses								
▪ Outcome measurec)	– BDI or BDI-II	3	0.28	0.10, 0.47	3.02**	0	0.94	6.41
	– Other	4	0.27	0.01, 0.53	2.06*	63.62*		6.58
▪ Control groupd)	– CAU	4	0.23	0.06, 0.40	2.71**	0	0.50	7.69
	– Other	3	0.34	0.06, 0.62	2.40*	65.28		5.26
▪ Personal contact	– Yes	3	0.16	−0.01, 0.34	1.82	0	0.095	11.11
	– No	4	0.38	0.20, 0.56	4.07***	23.73		4.72

*: p<0.05;

**: p<0.01;

***: p<0.001.

a) The p-value in this column indicates whether the Q statistic was significant or not.

b) This p-value indicates whether the effect sizes between subgroups differ significantly from each other.

c) The studies in which the BDI or BDI-II were also the studies that were conducted in Europe, while the studies using other instruments were conducted in the US.

d) The studies that did not use a care-as-usual control condition were the same studies that recruited patients from the general population.

Neither the funnel plot nor Duval and Tweedie's trim and fill procedure pointed at a significant publication bias. The effect size indicating the difference between the treatment and control condition and the effect size after adjustment for publication bias were exactly the same.

There were considerable differences between the characteristics of the studies. Therefore, we conducted a series of meta-analyses in which one of the studies was removed each time. Removal of the study by Salkovskis and colleagues [13] resulted in the largest increase of the effect size (the resulting effect size was 0.31; 95% CI: 0.18, 0.45; p<0.01); with *I^2^* = 17.63). After the removal of this study, we repeated this procedure and found that the study by De Graaf and colleagues [14] resulted in the largest increase of the effect size (d = 0.35; 95% CI: 0.21, 0.49; p<0.001; *I^2^* = 8.66%). We repeated these analyses, but this time we did not examine which studies contributed to an increase in the effect size, but to a decrease. This resulted in removal of the study of Meyer and colleagues [11] (d = 0.22; 95% CI: 0.09, 0.35 p<0.01; *I^2^* = 0), and Clarke and colleagues [35] (*d* = 0.21; 95% CI: 0.07, 0.36; p<0.01; *I^2^* = 0). These analyses did not result in clear indications that removal of individual studies resulted in important changes in mean effect size.

### Subgroup analyses

Because the number of studies was very small we conducted only the most basic subgroup analyses (Table 2). As can be seen, we found no indication for a significant difference between studies in which the BDI or BDI-II was used as an outcome measure and the studies in which other outcome measures were used. The studies in which the BDI or BDI-II were used, were also the studies that were conducted in Europe, while the studies using other instruments were conducted in the US. We also found no indication that studies in which care-as-usual control groups were used resulted in different effect sizes than studies in which another type of control group was used. The studies that did not use a care-as-usual control condition were the same studies that recruited patients from the general population. We found no significant difference between studies in which there was some personal contact at baseline and those without any contact. The results of these analyses have to be interpreted with caution because of the small number of studies per subgroup and because the characteristics overlapped considerably.

### Effects at longer-term follow-up

Six of the seven studies reported outcomes compared to the control group after post-test at longer-term follow-up (one did not [11]). The longer-term follow-up periods ranged from 4 to 12 months. The mean effect size indicating the difference between self-guided psychological treatment and control groups at four to six months follow-up was *d* = 0.18 (95% CI: −0.01,0.37; n.s.) with zero heterogeneity (*I^2^* = 0). At eight to twlve months follow-up the effect size was d = 0.27 (95% CI: 0.10, 0.44; p<0.01).

## Discussion

In this meta-analysis we found evidence that self-guided psychological treatment has a small but statistically significant effect on participants with elevated levels of depressive symptomatology. At four to twelve months these effects were still significant. Heterogeneity was low in the analyses, and there was no indication of publication bias. Although this finding is based on a relatively small number of studies, they all had relatively large sample sizes and were of high quality apart from the fact that diagnosis of depression was not established. Most individual studies did not have sufficient statistical power to detect a significant effect, but after pooling of the studies the effect size became highly significant.

The NNT of self-guided psychological treatment was between six and seven, indicating that one in every six or seven patients will benefit from such an intervention. Although this may not seem very high, comparable effect sizes NNTs are found in high-quality studies of face-to-face psychotherapies for depression [37]. Furthermore, a system without a coach or therapist does not require a complex and costly structure of professionals, and there is virtually no limit as to how many patients can enter the program, since additional patients will not imply additional therapist time.

The effect size we found was somewhat higher than the one that was found in an earlier meta-analysis of self-help (d = 0.06) [1]. This may be caused by the broader inclusion criteria used in that study, which also included trials focused on stress-related problems and anxiety.

While most studies found small, but positive effect sizes, we found one study that resulted in a small negative result for self-guided treatment [13]. Although heterogeneity was low, and the small effect size in this study may have been a chance finding, it is also possible that other reasons caused this deviation from the other studies. This was the only study in which bibliotherapy with a book was examined, while the other studies examined internet interventions. Furthermore, it was also the only study that was conducted among depressed GP patients. The other studies all recruited patients from other samples.

This study has several limitations. First, the number of included studies was small, although the included studies had a high methodological quality and large sample sizes. This small number of studies limited the possibilities to examine potential moderators of outcome. Second, there were considerable differences between the included studies. Only one study examined self-guided psychological treatment in book format [13], while the others examined Internet-based interventions. This one study found an effect size of about zero, leaving open the possibility that self-guided therapy is only effective in Internet-based format and not in the book format. This was also the only study in which patients had to meet diagnostic criteria for a depressive disorder. The small number of studies, however, does not allow us to examine whether this is indeed true. Third, while most studies in this meta-analysis included people with an elevated level of depressive symptoms, most did not establish the presence of a depressive disorder with a standardized diagnostic interview. Fourth, outcomes were not assessed by blind outcome assessors, but by self-report instruments. Since patients were not blinded, this may have introduced bias.Our findings do not imply that self-guided psychological treatment can be used in all patients seeking help in mental health care or primary care. The studies examined in this meta-analysis only included patients who were willing to be randomized to a self-guided therapy condition. People who were not interested in self-guided therapy probably did not participate in these trials, which likely resulted in sample bias. This means that self-guided therapy may indeed be effective for some people, but certainly not for all people with depression. Future research should examine who is willing to participate in self-guided psychological and who is not, and if there are differential predictors and mediators of outcome. Another related limitation is that studies fail to report negative outcomes of self-guided psychological treatment. However, there is little empirical support for the notion the self-help in general has unintended harmful effects [38], but this needs to be investigated further.

Despite these limitations, this meta-analysis has found considerable evidence that self-guided psychological treatment has a small effect on symptoms of depression. We could not establish that unguided psychological treatments work for patients with diagnosed major depression, but even subthreshold symptoms of depression can be treated and hopefully prevent the development of a depressive episode. It may be possible to integrate self-guided psychological treatments as a first step in stepped-care models for depression [39]. Such models often use watchful waiting as a first step, because many depressive symptoms improve spontaneously [40], [41]. Offering self-guided therapy, rather than watchful waiting, as a first step would be easy to implement and may well improve outcomes. For patients who do not respond to self-guided therapy (which will be the case for most patients), more intensive treatment can be initiated as the second step of the program. Moreover, with the recent development of Internet-delivered interventions it may be that automated systems can mimic the role of the therapist and increase the effects of unguided treatment. It could also be that the treatment materials used in unguided treatments could be improved by adding elements to boost the therapeutic elements like the therapeutic alliance.

Another important issue in self-guided treatment is adherence. Although the attrition rates from the studies we found do not differ very much from the rates found in psychotherapy studies in general, the actual use of the treatments by patients was low. For example, in one of the studies it was found that 38% of the patients did not complete the first session, and only 14% completed all sessions [14]. This could be a problem that is aggravated in depression, since anhedonia and loss of motivation are cardinal symptoms of depression. This may suggest that self-guided treatments are especially effective in patients who are very motivated for this kind of treatment and are capable of finishing the intervention without stimulation from an external therapist or coach.

This study showed that self-guided psychological treatment has a small but significant effect on participants with increased levels of depressive symptomatology. The next step is to examine whether such interventions can be implemented in routine practice in a stepped care model and whether the effects found in this meta-analysis will be found in regular clinical settings. We also encourage more research on predictors of outcome and reports of both responders and non-responders to unguided psychological treatment.

## Supporting Information

Appendix S1 Full search string for PubMed.
(DOC)

## References

[1] Gellatly J, Bower P, Hennessy S, Richards, Gilbody S (2007). What makes self-help interventions effective in the management of depressive symptoms? Meta-analysis and meta-regression.. Psychol Med.

[2] Gregory R, Canning S, Lee T, Wise J (2004). Cognitive Bibliotherapy for Depression: A Meta-Analysis.. Prof Psychol Res Pract.

[3] Menchola M, Arkowitz HS, Burke BL (2007). Efficacy of self-administered treatments for depression and anxiety.. Prof Psychol Res Pract.

[4] Cuijpers P (1997). Bibliotherapy in unipolar depression.. J Behav Ther Exp Psychiatry.

[5] Hirai M, Clum GA (2006). A meta-analytic study of self-help interventions for anxiety problems.. Behav Ther.

[6] Spek V, Cuijpers P, Nyklíček I, Riper H, Keyzer J (2007). Internet-based cognitive behavior therapy for mood and anxiety disorders: a meta-analysis.. Psychol Med.

[7] Van Straten A, Cuijpers P (2009). Self-help therapy for insomnia: a meta-analysis.. Sleep Med Rev.

[8] Haddock CK, Rowan AB, Andrasik F, Wilson PG, Talcott GW (1997). Home-based behavioral treatments for chronic benign headache: A meta-analysis of controlled trials.. Cephalagia.

[9] Marrs RW (1995). A meta-analysis of bibliotherapy studies.. Am J Commun Psychol.

[10] Cuijpers P, Donker T, van Straten A, Li J, Andersson G (2010). Is guided self-help as effective as face-to-face psychotherapy for depression and anxiety disorders? A systematic review and meta-analysis of comparative outcome studies.. Psychol Med.

[11] Meyer B, Berger T, Caspar F, Beevers CG, Andersson G (2009). Effectiveness of a novel integrative online treatment for depression (Deprexis): Randomized controlled trial.. J Med Internet Res.

[12] Spek V, Nyklíček I, Smits N, Cuijpers P, Riper H (2007). Internet-based cognitive behavioural therapy for sub-threshold depression in people over 50 years old: A randomized controlled clinical trial.. Psychol Med.

[13] Salkovskis P, Rimes K, Stephenson D, Sacks G, Scott J (2006). A randomized controlled trial of the use of self-help materials in addition to standard general practice treatment of depression compared to standard treatment alone.. Psychol Med.

[14] de Graaf LE, Gerhards SA, Arntz A, Riper H, Metsemakers JF (2009). Clinical effectiveness of online computerised cognitive-behavioural therapy without support for depression in primary care: randomised trial.. Br J Psychiatry.

[15] Andersson G, Cuijpers P (2009). Internet-based and other computerized psychological treatments for adult depression: A meta-analysis.. Cogn Behav Therap.

[16] Cuijpers P, van Straten A, Warmerdam L, Andersson G (2008). Psychological treatment of depression: A meta-analytic database of randomized studies.. BMC Psychiatry.

[17] Christensen H, Griffiths KM, Jorm A (2004). Delivering interventions for depression by using the internet: randomised controlled trial.. BMJ.

[18] Patten SB (2003). Prevention of depressive symptoms through the use of distance technologies.. Psychiatry Serv.

[19] Little P, Dorward M, Warner G, Moore M, Stephens K Randomised controlled trial of effect of leaflets to empower patients in consultations in primary care.. BMJ.

[20] Webster J, Linnane J, Roberts J, Starrenburg S, Hinson J (2003). IDentify, Educate and Alert (IDEA) trial: an intervention to reduce postnatal depression.. BJOG.

[21] Rahe RH, Taylor CB, Tolles RL, Newhall LM, Veach TL (2002). A novel stress and coping workplace program reduces illness and healthcare utilization.. Psychosom Med.

[22] Zetterqvist K, Maanmies J, Ström L, Andersson G (2003). Randomized controlled trial of internet-based stress management.. Cogn Behav Ther.

[23] Grime PR (2004). Computerized cognitive behavioural therapy at work: a randomized controlled trial in employees with recent stress-related absenteeism.. Occup Med.

[24] Fletcher J, Lovell K, Bower P, Campbell M (2005). Process and outcome of a non-guided self-help manual for anxiety and depression in primary care: A pilot study.. Behav Cogn Psychother.

[25] Holdsworth N, Paxton R, Seidel S, Thomson D, Shrubb S (1996). Paralled evaluations of new guidance materials for anxiety and depression in primary care.. J Mental Health.

[26] Higgins JPT, Green S (2008). Cochrane Handbook for Systematic Reviews of Interventions Version 5.0.1 [updated September 2008]. The Cochrane Collaboration.. http://www.cochrane-handbook.org.

[27] Cohen J (1988). Statistical power analysis for the behavioral sciences (2nd ed.).

[28] Kraemer HC, Kupfer DJ (2006). Size of treatment effects and their importance to clinical research and practice.. Biol Psychiatry.

[29] Sackett DL, Strauss SE, Richardson WS, Rosenberg W, Haynes RB (2000). Evidence-based medicine. How to practice and teach EBM. 2 ed.

[30] Higgins JP, Thompson SG, Deeks JJ, Altman DG (2003). Measuring inconsistency in meta-analyses.. BMJ.

[31] Duval S, Tweedie R (2000). Trim and fill: A simple funnel-plot-based method of testing and adjusting for publication bias in meta-analysis.. Biometr.

[32] Borenstein M, Hedges LV, Higgins JPT, Rothstein HR (2009). Introduction to Meta-Analysis.

[33] Christensen H, Griffiths KM, Mackinnon AJ, Brittliffe K (2006). Online randomized controlled trial of brief and full cognitive behaviour therapy for depression.. Psychol Med.

[34] Clarke G, Reid E, Eubanks D, O'Connor E, DeBar LL (2002). Overcoming depression on the Internet (ODIN): a randomized controlled trial of an Internet depression skills intervention program.. J Med Internet Res.

[35] Clarke G, Eubanks D, Reid E, Kelleher C, O'Connor E, DeBar LL (2005). Overcoming Depression on the Internet (ODIN) (2): A randomized trial of a self-help depression skills program with reminders.. J Med Internet Res.

[36] Clarke G, Kelleher C, Hornbrook M, Debar L, Dickerson J (2009). Randomized effectiveness trial of an Internet, pure self-help, cognitive behavioral intervention for depressive symptoms in young adults.. Cogn Behav Ther.

[37] Cuijpers P, van Straten A, Bohlmeijer E, Hollon SD, Andersson G (2010). The effects of psychotherapy for adult depression are overestimated: A meta-analysis of study quality and effect size.. Psychol Med.

[38] Scogin F, Floyd M, Jamison C, Ackerson J, Landreville P (1996). Negative outcomes: What is the evidence on self-administered treatments?. J Consult Clin Psychol.

[39] Van Straten A, Seekles W, van 't Veer-Tazelaar N, Beekman ATF (2010). Stepped care for depression in primary care: what should be offered and how?. Med J Austr.

[40] Seekles W, van Straten A, Beekman A, van Marwijk H, Cuijpers P (2009). Stepped care for depression and anxiety: a randomised controlled trial testing the effectiveness of a stepped care program among primary care patients with mood or anxiety disorders.. BMC Health Serv Res.

[41] Van 't Veer-Tazelaar PA, van Marwijk HWJ, van Oppen P, van Hout HPJ, van der Horst HE (2009). Stepped-care prevention of anxiety and depression in late life: a randomized controlled trial.. Arch Gen Psychiatry.

