# Supplementary material for: Self-Guided Psychological Treatment for Depressive Symptoms: A Meta-Analysis
Source: PLoS One. 2011 Jun 21;6(6):e21274. doi: 10.1371/journal.pone.0021274 (PMC3119687; doi:10.1371/journal.pone.0021274)
Supplement: Appendix S1 — Full search string for PubMed. (DOC) [file pone.0021274.s001.doc]

Appendix S1. Full search string for PubMed

(("behaviour therapy"[All Fields] OR "behavior therapy"[MeSH Terms] OR ("behavior"[All Fields] AND "therapy"[All Fields]) OR "behavior therapy"[All Fields]) OR ("biofeedback, psychology"[MeSH Terms] OR ("biofeedback"[All Fields] AND "psychology"[All Fields]) OR "psychology biofeedback"[All Fields] OR "biofeedback"[All Fields]) OR (cognitive[All Fields] AND analytic[All Fields] AND ("therapy"[Subheading] OR "therapy"[All Fields] OR "therapeutics"[MeSH Terms] OR "therapeutics"[All Fields])) OR ("cognitive behaviour therapy"[All Fields] OR "cognitive therapy"[MeSH Terms] OR ("cognitive"[All Fields] AND "therapy"[All Fields]) OR "cognitive therapy"[All Fields] OR ("cognitive"[All Fields] AND "behavior"[All Fields] AND "therapy"[All Fields]) OR "cognitive behavior therapy"[All Fields]) OR ("counselling"[All Fields] OR "counseling"[MeSH Terms] OR "counseling"[All Fields]) OR ("family therapy"[MeSH Terms] OR ("family"[All Fields] AND "therapy"[All Fields]) OR "family therapy"[All Fields]) OR ("marital therapy"[MeSH Terms] OR ("marital"[All Fields] AND "therapy"[All Fields]) OR "marital therapy"[All Fields]) OR ("psychoanalytic therapy"[MeSH Terms] OR ("psychoanalytic"[All Fields] AND "therapy"[All Fields]) OR "psychoanalytic therapy"[All Fields]) OR ("psychotherapy"[MeSH Terms] OR "psychotherapy"[All Fields]) OR ("relaxation therapy"[MeSH Terms] OR ("relaxation"[All Fields] AND "therapy"[All Fields]) OR "relaxation therapy"[All Fields])) AND (("depressive disorder"[MeSH Terms] OR ("depressive"[All Fields] AND "disorder"[All Fields]) OR "depressive disorder"[All Fields] OR "depression"[All Fields] OR "depression"[MeSH Terms]) OR depressive[All Fields]) OR ("dysthymic disorder"[MeSH Terms] OR ("dysthymic"[All Fields] AND "disorder"[All Fields]) OR "dysthymic disorder"[All Fields] OR "dysthymia"[All Fields])
